# Dietary Inclusion of Crickets (*Acheta domesticus*) and Yellow Mealworm Meal (*Tenebrio molitor*) in Comparison with Soybean Meal: Effect on the Growth, Total Tract Apparent Digestibility, and Nitrogen Balance of Fattening Rabbits

**DOI:** 10.3390/ani13101637

**Published:** 2023-05-14

**Authors:** Zdeněk Volek, Lukáš Zita, Anna Adámková, Martin Adámek, Jiří Mlček, Vladimír Plachý

**Affiliations:** 1 Department of Nutritional Physiology and Animal Product Quality, Institute of Animal Science, Přátelství 815, 104 00 Prague, Czech Republic; volek.zdenek@vuzv.cz; 2 Department of Microbiology, Nutrition and Dietetics, Faculty of Agrobiology, Food and Natural Resources, Czech University of Life Sciences Prague, 165 00 Prague, Czech Republic; plachy@af.czu.cz; 3 Department of Animal Science, Faculty of Agrobiology, Food and Natural Resources, Czech University of Life Sciences Prague, 165 00 Prague, Czech Republic; zita@af.czu.cz; 4 Department of Food Analysis and Chemistry, Faculty of Technology, Tomas Bata University in Zlín, Vavrečkova 5669, 760 01 Zlín, Czech Republic; aadamkova@utb.cz; 5 Department of Automation and Control Engineering, Faculty of Applied Informatics, Tomas Bata University in Zlín, Nad Stráněmi 4511, 760 05 Zlín, Czech Republic; m2adamek@utb.cz; 6 Department of Microelectronics, Faculty of Electrical Engineering and Communication, Brno University of Technology, Technická 3058/10, 616 00 Brno, Czech Republic

**Keywords:** rabbit, diet, crude protein, insect meal, digestibility, nitrogen output

## Abstract

**Simple Summary:**

There is a predicted increase in the world population by 2050, and the main task will be to ensure sufficient safe food. In the situation of food–feed–biofuel competition, a circular economy can represent a smart solution. The utilization of agricultural/food wastes and byproducts by insects to gain quality feed components, such as insect meals, for monogastric animals seems to be one suitable solution. In this study, we evaluated the effect of diets based on insect meal (adult *Acheta domesticus*, AD, and *Tenebrio molitor* larvae, TM) in comparison with soybean meal (SM) on the coefficient of the total tract apparent digestibility of the experimental diets, the nitrogen balance, and the growth performance of rabbits. Based on the results of this study, it can be concluded that both insect meals tested (AD and TM) are suitable dietary crude protein sources that can fully replace SM (currently criticized because of deforestation) for growing-fattening rabbits. Regarding nitrogen output, it seems that the dietary inclusion of AD in rabbit diets might have better potential in terms of an environmental impact than the dietary inclusion of TM because of the higher essential amino acid content in AD.

**Abstract:**

Three diets were formulated, i.e., soybean meal (SM diet), adult *Acheta domesticus* (AD diet), and *Tenebrio molitor* larvae (TM diet), as the main crude protein (CP) sources. A total of 45 rabbits (Hyplus, weaned at 32 days of age) were divided into three groups (15 per treatment) and fed one of the three diets for 42 days. A higher daily weight gain (*p* = 0.042), as well as daily feed intake (*p* = 0.022), was observed in rabbits fed the AD and TM diets than in rabbits fed the SM diet within 21 days after weaning. The coefficients of total tract apparent digestibility (CTTAD) of gross energy were higher (*p* = 0.001) in rabbits fed the SM diet than in rabbits fed the other diets. The CTTAD of CP (*p* = 0.040) and starch (*p* = 0.041) was higher in rabbits fed the SM diet compared to those fed the AD diet. There were non-significantly higher losses of nitrogen in the urine (an average by 0.227 g/day; *p* = 0.094) in rabbits fed the TM diet than in rabbits fed the other diets. It can be concluded that the growth of rabbits and nitrogen output were not detrimentally affected by the insect meal (AD or TM) used in this study.

## 1. Introduction

The world population is predicted to grow to 9.8 billion by 2050 [1]. To provide enough food for this increased world population, the agri-food sector has to solve the problems of significant food losses and food waste in terms of a whole chain between farms, food processing, and consumers [2]. In general, food security is a crucial task of a total of 17 Sustainable Development Goals of the United Nations [3]. In this respect, the utilization of agricultural/food wastes and byproducts in animal nutrition, as a tool of a circular economy to reduce the negative impact of the food production chain on the environment, might represent a sustainable solution [4,5].

From a circular economy and the impact of agriculture on the environment point of view, rabbit meat production might offer an interesting opportunity to contribute to the increasing global demand for quality foods. It is known that most of the coproducts of agro-industrial production contain abundant dietary fiber, and rabbits are an ideal animal for the utilization of these products. In fact, dietary fiber represents the principal fraction of rabbit diets [6]. As far as the dietary utilization of byproducts in rabbits is concerned, a list of different products from agro-industrial processes may be drafted. It has been reported that coproducts, such as wheat bran [7], sugar beet pulp [8], sunflower hulls [9], soybean hulls [10], lupine hulls [11], lupine bran [12], soybean meal and sunflower meal [13], rapeseed meal [14], distillers dried grains with solubles [15], dried brewers’ grains [16], coproducts from olive cake [17], citrus coproducts [18], and defatted grape seed meal [19], can be suitable diet components for rabbits.

In addition to plant-based coproducts of agro-industrial production, it seems that rabbits are also able to utilize feed components of animal coproducts (blood meal, enzymatically digested feather meal, fish meal, leather hydrolysates, meat meal, dried skimmed milk, dried or fresh whey, bone meal, etc.) [20,21]. Most of these products were experimentally tested as protein sources, whereas fresh whey or dried whey was the source of lactose (energy) [20]. Similarly, insect meals are increasingly considered a valuable source of nutrients and energy for farmed animals [21,22]. As the sustainability of soy production as a sustainable value chain is increasingly criticized (because of deforestation and the carbon footprint), nutritionists now have a concentrated focus, among others, on replacing the feed ingredient soy with insects [22].

Regarding rabbit nutrition, encouraging results have been reported in terms of the effect of the dietary inclusion of silkworm pupae meal (*Bombyx mori*), maggot meal (*Musca domestica*, larvae), and yellow mealworm larvae (*Tenebrio molitor*, TM). It was observed that insect meal in the rabbit diet did not detrimentally affect the growth performance [23,24,25,26,27,28,29], feed efficiency [23,28], nutrient digestibility and nitrogen output [27,28], gastrointestinal function [27], and rabbit meat and carcass quality [25]. Thus, there are reports in the literature that mealworm larvae meal, silkworm pupae meal, and housefly pupae meal can be utilized in a rabbit diet [21].

To our knowledge, however, there is scant information in the literature concerning other insect species (e.g., crickets, *Acheta domesticus*, AD) in terms of rabbit nutrition. Based on the abovementioned studies regarding TM, maggot meal or silkworm pupae meal, a hypothesis can be proposed that crickets can serve as a suitable alternative for soybean meal (SM) in diets for rabbits after weaning. 

Thus, the present study aimed to extend our current knowledge by obtaining novel information concerning the comparison of two types of insect meal (*Acheta domesticus* and *Tenebrio molitor*) with SM in terms of the effect of diets based on insect meal on the coefficient of total tract apparent digestibility (CTTAD) of the experimental diets, nitrogen excretion, and the growth performance of rabbits after weaning.

## 2. Materials and Methods

### 2.1. Soybean Meal, Acheta Domesticus, Tenebrio Molitor, and Feed Mixtures

The chemical compositions of the soybean meal, *Acheta domesticus,* and *Tenebrio Molitor* tested are presented in Table 1. *Tenebrio molitor* larvae meal and adult *Acheta domesticus* meal were purchased from Papek s.r.o. (Jaroměřice nad Rokytnou, Czech Republic). As feed for AD and TM, potatoes without the peel, fruit, and vegetables were used. Cereal by-products and cereal meal were used as litter. Insects used in this study had a higher level of crude protein, essential amino acids (EAAs), fiber, and ether extract than soybean meal, whereas the levels of calcium, phosphorus, and ash were lower.

For this study, three diets were formulated with different main crude protein sources, i.e., 60.00 g/kg SM (SM diet), 30.00 g/kg AD (AD diet), and 38.50 g/kg TM (TM diet) (Table 2). Soybean meal was fully replaced by AD and TM in the AD and TM diets, respectively. Other than the main crude protein sources, the diets had a similar basal mixture of ingredients. No additional fats or synthetic amino acids (AA) were used in the formulation of the diets. The diets did not differ in terms of crude protein (CP), starch, ether extract (EE), or ash. Additionally, the digestible protein/digestible ratio was designed to be similar for all diets. The diets differed slightly in the acid detergent fiber (ADF)/starch ratio. The SM diet had a lower content of ADF than the AD and TM diets, whereas the TM diet had the lowest level of EAAs. The diets were pelleted (3 mm × 5–10 mm length). No restrictions were implicated in terms of access of rabbits to feed and water during the fattening period. The diets used in the present study were not supplemented with a coccidiostatic additive or antibiotics.

**Table 2:** Components and chemical composition (g/kg on an as-fed basis unless otherwise stated) of the experimental diets based on soybean meal (SM diet), crickets (*Acheta domesticus*, AD diet), and yellow mealworm (*Tenebrio molitor*, TM diet).

Ingredient	SM Diet	AD Diet	TM Diet
Alfalfa meal	300.0	300.0	300.0
Soybean meal	60.00	0	0
Crickets	0	30.00	0
Yellow mealworm	0	0	38.50
Wheat bran	330.0	360.0	351.5
Sugar beet pulp	70.00	70.00	70.00
Oats	150.0	150.0	150.0
Barley	60.00	60.00	60.00
Vitamin–mineral supplement ^1^	10.00	10.00	10.00
Mono-calcium phosphate	5.00	5.00	5.00
Limestone	10.00	10.00	10.00
Salt	5.00	5.00	5.00
Analyzed composition			
Dry matter	872.1	870.2	879.4
Crude protein	169.3	167.4	165.1
aNDFom ^2^	359.4	357.3	390.3
ADFom ^3^	165.2	185.2	189.1
Starch	172.2	164.3	170.3
ADF/starch ratio	0.959	1.127	1.110
Ether extract	24.32	27.09	34.15
Lysine	7.40	7.39	7.11
Methionine	3.29	3.30	3.24
Threonine	5.60	5.69	5.30
Arginine	8.69	9.09	8.22
Tryptophan	15.19	14.39	14.69
Valine	7.79	8.09	7.29
Leucine	10.29	10.59	9.30
Isoleucine	5.79	6.10	5.29
Histidine	4.01	4.19	3.89
Phenylalanine	6.70	6.59	5.69
Essential amino acids total	74.75	75.42	70.02
Ash	73.12	73.06	72.15
Gross energy (MJ/kg)	15.79	16.10	16.30
Calculated value ^4^			
Digestible energy (MJ/kg)	10.50	10.03	9.88
Digestible protein	129.2	122.0	124.2
Digestible protein/digestible energy ratio (g/MJ)	12.31	12.16	12.57

^1^ Per kilogram of complete feed mixture: vitamin A (retinol), 12,000 IU; vitamin D3 (cholecalciferol), 2000 IU; vitamin E (α-tocopherol), 50.00 mg; vitamin K3 (bisulfite menadione complex), 2.00 mg; vitamin B1 (thiamine mononitrate), 3.00 mg; vitamin B2 (riboflavin), 7.00 mg; vitamin B6 (pyridoxine), 4.00 mg; niacinamide, 50.00 mg; Ca-pantothenate, 20.00 mg; folic acid, 1.70 mg; biotin, 0.200 mg; vitamin B12 (cyanocobalamin), 0.020 mg; choline chloride, 600.0 mg; Cu (as CuSO_4_·5H_2_O), 15.00 mg; Fe (as FeSO_4_·7H_2_O), 50.00 mg; I (as KI), 1.2 mg; Mn (as MnO), 47.00 mg; Zn (as ZnO), 50.00 mg; and Se (as Na_2_SeO_3_). ^2^ Neutral detergent fiber (treated with a heat-stable amylase, expressed excluding residual ash). ^3^ Acid detergent fiber expressed without residual ash. ^4^ The digestible energy and digestible protein contents of the diets tested in the present study were calculated from digestibility coefficients (Table 3).

**Table 3:** Live weight, average daily weight gain, average daily feed intake, the feed conversion ratio of rabbits (the entire period; 32 to 74 days of age), coefficients of total tract apparent digestibility (CTTAD), and N-balance, and N-retention in rabbits fed diets based on soybean meal (SM diet), yellow mealworm (*Tenebrio molitor*, TM diet) or adult crickets (*Acheta domesticus*, AD diet).

Traits	SM Diet	TM Diet	AD Diet	SEM	*p* Value
Live weight ^1^ (g)					
at 32 days of age ^2^	796.1	822.3	832.2	19.5	0.760
at 53 days of age	1846	1989	2025	39	0.152
at 74 days of age	2802	2892	3027	52	0.228
Average daily weight gain (g)					
32–53 days of age	50.00 ^b^	55.60 ^a^	56.78 ^a^	1.16	0.042
53–74 days of age	45.50	43.00	47.73	1.07	0.183
32–74 days of age	47.75	49.30	52.25	0.95	0.155
Average daily feed intake (g)					
32–53 days of age	112.3 ^b^	129.8 ^a^	133.2 ^a^	3.3	0.022
53–74 days of age	159.6	162.3	175.4	4.1	0.271
32–74 days of age	135.9	148.0	154.8	3.5	0.092
Feed conversion ratio					
32–53 days of age	2.25	2.35	2.35	0.04	0.923
53–74 days of age	3.51	3.77	3.68	0.08	0.125
32–74 days of age	2.85	3.00	2.96	0.03	0.139
Mortality (%)	0	0	0	-	-
Morbidity (%)	0	0	0	-	-
CTTAD ^3^					
Organic matter	0.676 ^a^	0.616 ^b^	0.628 ^b^	0.007	0.001
Crude protein	0.763 ^a^	0.752 ^ab^	0.729 ^b^	0.006	0.040
Starch	0.974 ^a^	0.968 ^ab^	0.966 ^b^	0.001	0.041
Acid detergent fiber	0.299	0.259	0.280	0.012	0.231
Gross energy	0.665 ^a^	0.606 ^b^	0.623 ^b^	0.006	0.001
Nitrogen balance ^3^ (g/day)					
Nitrogen intake	4.07	4.35	4.28	0.10	0.549
Nitrogen excretion in feces	0.976	1.09	1.17	0.043	0.215
Nitrogen excretion in urine	0.621	0.884	0.693	0.051	0.094
Total nitrogen excretion	1.60	1.97	1.86	0.08	0.131
Nitrogen retention					
Retained nitrogen ^4^ (g/day)	2.48	2.38	2.42	0.06	0.812
Coefficient of nitrogen retention ^5^	0.608	0.555	0.563	0.011	0.099

^1^ 15 rabbits (males) per diet (rabbits were individually housed using metabolic wire-net cages); ^2^ At weaning; ^3^ Determined in rabbits between 50 and 54 days of age; ^4^ nitrogen intake-total nitrogen excretion (feces + urine); ^5^ retained nitrogen/nitrogen intake. ^a,b^ Within a row, different superscripts indicate significant differences between means (*p* < 0.05).

### 2.2. Rabbits and Experimental Design

The study was performed in the rabbit facility of the Czech University of Life Sciences, Prague (Prague, Czech Republic) (the Demonstration and Experimental Center of the Faculty of Agrobiology, Food and Natural Resources), which is accredited with European Union Standards. The rabbits were kept in an air-conditioned room equipped with a forced ventilation system (the temperature inside the room was 16 °C, the relative humidity inside the room was 65%, and we used a 12 h/d light program). This study was approved by the ethics board of the Czech University of Life Science Prague (Expert commission ensuring the welfare of experimental animals).

For the purpose of this study, a total of 45 Hyplus rabbits (PS 19 × PS 40, males, at 32 days of age, after weaning) were used for the evaluation of the CTTAD of organic matter (OM), CP, starch, ADF, and gross energy (GE) of the diets tested and to determine the nitrogen retention and nitrogen balance. To determine the CTTAD of the diets, an international method was used [30]. During the whole period of fattening (i.e., between 32 and 74 days of age), data concerning the growth performance of rabbits were also recorded. Rabbits were divided into three groups (15 per treatment) and fed one of the three diets ad libitum for 42 days. Animals were housed in wire net metabolic cages (50 × 40 × 42.5 cm; one rabbit per cage). The feed intake of rabbits was checked daily, whereas the live weight of animals was recorded in weekly intervals. Based on these data, the average daily feed intake (FI), average daily weight gain (ADG), and feed conversion ratio (FCR) were calculated. Health status (mortality and morbidity) was checked by means of individual observation of clinical signs of digestive problems according to the methodology of the European Group on Rabbit Nutrition [31]. After an adaptation period of 18 days, the total individual fecal and urine output (between 50 and 54 days of age) was measured as described by others [32,33]. Briefly, every day (between 0800 and 0900 h), feces and urine were collected separately using a perforated plate for feces placed under the cage. Urine was collected in a beaker. The beaker was placed under the plate for feces collection. The beaker contained 40 mL of sulfuric acid (10%, *v*/*v*). These samples were stored at −18 °C for analysis. 

### 2.3. Chemical Analysis

Before analysis, samples of SM, AD, TM, the experimental feed mixture, and feces were processed using a 1 mm screen. All samples were analyzed in duplicate. The contents of dry matter (DM, method 934.01), ash (method 942.05), starch (method 920.40), ether extract (EE, method 920.39), CP (method 954.01), and ADF (method 973.18) were analyzed according to the procedures of AOAC International [34]. Nitrogen levels in samples of CP sources, feeds, urine, and feces and EE in the feed and CP sources were analyzed using common types of equipment (Kjeltec Auto 1030 Analyzer and Soxtec 1043, respectively; FOSS Tecator AB, Höganäs, Sweden). Neutral detergent fiber was treated with a heat-stable amylase and expressed excluding residual ash) (aNDFom) [35]. Gross energy was measured by combustion in an adiabatic calorimeter (C5000 control, IKA-Werke, Staufen, Germany). The AA contents in the diets and protein sources tested in the present study were determined as described by others [32]. Briefly, the isolation of amino acids was carried out by hydrolysis in 6 M hydrochloric acid at 110 °C for 23 h. In the case of the determination of sulfur amino acids, oxidation with peroxoformic acid was preceded by hydrolysis at a cold temperature for 16 h. After evaporation, the hydrolysate was dissolved in citrate buffer at pH 2.2 and diluted to a suitable concentration. In the case of tryptophan determination, hydrolysis was carried out in 4 M lithium hydroxide. The hydrolysate was neutralized with neutralizing citrate buffer and centrifuged at 10,000 RPM. Amino acids were analyzed using an AAA-400 amino acid analyzer (INGOS Ltd., Prague, Czech Republic). Analysis included separation on an ion exchange column using citrate buffers, postcolumn derivatization with ninhydrin reagent, and spectrophotometric detection at wavelengths of 440 and 570 nm. Chromatograms were evaluated based on comparison with an external standard. The P and Ca contents in SM, AD, and TM were determined by atomic absorption spectrometry performed using a Solaar M6 instrument (TJA Solutions, Cambridge, UK).

### 2.4. Statistical Analyses

The FI, ADG, FCR, CTTAD, nitrogen balance, and nitrogen retention were examined by one-way ANOVA using the GLM procedure in the Statistical Analysis System with diet type as the fixed effect (SAS Institute Inc., Cary, NC, USA, 2006). For the comparison of means, the Scheffe test was used. The individual rabbits represented the experimental unit for CTTAD, nitrogen intake, nitrogen excretion, and N-retention. Differences with *p* < 0.05 were considered significant, and all differences with *p* values between 0.05 and 0.10 were considered trends.

## 3. Results

### Growth Performance, CTTAD, and Nitrogen Output

The live weight of rabbits, FI, ADG, FCR, N-balance, and nitrogen retention are shown in Table 3. There was a higher ADG (on average by 6.19 g; *p* = 0.042) in rabbits fed the AD and TM diets compared to those fed the SM diet in the first three weeks after weaning. Similarly, FI was higher in rabbits fed the diets with insect meal compared to those fed the SM diet within the first three weeks after weaning (on average by 19.20 g; *p* = 0.022), and a tendency was observed for the whole period of fattening (on average by 15.50 g; *p* = 0.092). The FI between 53 and 74 days of age, ADG and FCR during the whole fattening period, and the live weight of rabbits at 53 days of age and at the end of the fattening period (74 days of age) were not affected by the dietary treatments. Regarding the health status of rabbits during the whole fattening period, no mortality or morbidity was recorded.

The CTTAD of OM and gross energy were significantly higher in rabbits fed the SM diet compared to those fed the AD or TM diet. The CTTAD of CP and starch was significantly higher in rabbits fed the SM diet compared to those fed the AD diet. No significant differences were observed with respect to the CTTAD of ADF.

Nitrogen intake and nitrogen excretion in feces were not affected by dietary treatments. There was a tendency for higher losses of nitrogen in the urine (on average by 0.227 g/day; *p* = 0.094) in rabbits fed the TM diet compared to those fed the other diets. Consequently, the nitrogen retention coefficient was lower in rabbits fed the TM diet (by an average of 0.031; *p* = 0.099) than in those fed the SM or AD diet.

## 4. Discussion

In the present study, the contents of CP, EE, ADFom, EAAs, Ca, P and ash in the main crude protein sources tested are similar to those reported by other authors [14,28,36,37]. In insects, as expected, protein was the nutrient in the highest concentration because the exoskeleton is rich in protein [36]. A low level of ash (especially calcium) was observed in the insect meals tested in the present study, a finding typically reported for insects [38]. In fact, in most insects, the skeleton is not sufficiently calcified [36]. In this study, we observed relatively high levels of ether extract, a finding which is usually observed in insects [37]. Indeed, fat is an abundant component of most insect species [36]. Regarding the ADFom content detected in the insect meal used in this study, there is an assumption that this fiber fraction likely represents both chitin and sclerotized protein [36].

A higher FI was observed in rabbits fed diets based on insect meal (AD and TM diets). As observed by others [38], this finding may be explained by a lower dietary energy content and a higher content of ADFom in these diets than in the SM diet. Appetite in rabbits is regulated by means of a chemostatic mechanism, resulting in daily energy ingestion being constant; thus, to cover daily energy requirements, the level of feed consumed by rabbits, to some extent, depends on the dietary digestible energy content [39]. Moreover, it is known that increasing the dietary fiber content also increases dry matter intake by rabbits [40]. The higher feed intake of rabbits fed the AD and TM diets was probably associated with the higher ADG observed in these rabbits. Similarly, a higher average daily weight gain, associated with a higher average daily feed intake, was observed by other authors [28]. In general, there was no detrimental effect of the insect meal tested in this study on growth performance, which is in accordance with the results of other studies [25,26,27,28]. There were no detrimental effects of the diets based on insect meal on the health status of rabbits, which is in line with results reported by other authors [28].

In this study, we observed a lower CTTAD of energy in rabbits fed diets containing insect meal compared to those fed the SM diet, a finding that is probably associated with a higher ADFom level in the diets based on insect meal. In this respect, there is a clear relationship between the dietary fiber content and gross energy digestibility [41]. If the fiber content of the feed increases, energy digestibility usually decreases. A lower CTTAD of starch was measured in rabbits fed the AD diet compared to those fed the SM diet. This finding may be due to a higher ADFom/starch ratio in the AD diet, which is in line with results reported by other authors [42,43]. There was a lower CTTAD of crude protein in rabbits fed the diet based on AD than in rabbits fed the SM diet, a finding that is probably associated with the type of feed. Indeed, it is assumed that protein digestibility varies more according to the type of feed ingredient than to the chemical composition [13]. As a consequence of a lower CTTAD of CP and starch, we observed a lower CTTAD of organic matter in rabbits fed the diets based on insect meal than in those fed the SM diet.

In the present study, on average, losses of nitrogen in feces accounted for 25% of the nitrogen intake (24, 25, and 27% for rabbits fed the SM, TM, and AD diets, respectively), and this finding is in line with results reported by other authors [28,32,44]. The proportion of nitrogen that is excreted by feces is related to the apparent digestibility of the crude protein, recorded for the entire period of fattening [44]. Higher losses of nitrogen in the urine (45% of the total nitrogen excretion) were observed in rabbits fed the TM diet compared to those fed the SM and AD diets (39% and 37% of the total excretion of nitrogen, respectively). Consequently, the coefficient of N-retention was lower in rabbits fed the TM diet. This finding is apparently caused by a lower content of EAAs in the TM diet than in the SM and AD diets.

## 5. Conclusions

Based on the results of this study, we conclude that the dietary inclusion of insect meal (*Acheta domesticus*, AD, or *Tenebrio molitor*, TM) is a suitable replacement for soybean meal in rabbit diets. The growth performance of rabbits, their health and nitrogen balance, and nitrogen outputs were not detrimentally affected by the dietary insect meal used in this study. Regarding nitrogen output, it seems that the dietary inclusion of AD in rabbit diets might have better potential in terms of an environmental impact than the dietary inclusion of TM because of a higher essential amino acid content in AD. This aspect, as well as the economic viability of using this type of ingredient, should be studied in further experiments.

## Figures and Tables

**Table 1:** Chemical composition of soybean meal, *Acheta domesticus,* and *Tenebrio molitor* larvae meal (g/kg on an as-fed basis).

Item	Soybean Meal	*Acheta* *domesticus*	*Tenebrio molitor* Larvae Meal
Dry matter	883.2	922.1	971.2
Crude protein	469.4	642.2	531.3
ADFom ^1^	66.18	99.32	152.2
Ether extract	20.14	186.4	307.3
Lysine	26.29	35.59	28.09
Methionine	6.60	9.40	6.69
Threonine	17.09	21.89	18.80
Arginine	32.49	38.59	26.29
Tryptophan	6.30	29.20	32.89
Valine	21.29	33.59	29.79
Leucine	33.11	47.29	37.90
Isoleucine	20.69	30.60	27.79
Histidine	11.40	15.29	16.49
Phenylalanine	22.49	20.39	15.50
Total essential amino acids	197.8	281.8	240.2
Ca	3.32	2.41	1.21
P	6.52	5.83	12.12
Ash	62.24	46.12	48.14

^1^ Acid detergent fiber content is expressed excluding residual ash.

## Data Availability

The data, presented in this study, are available by reasonable request from the corresponding author.
